# On the accuracy of dose prediction near metal fixation devices for spine SBRT

**DOI:** 10.1120/jacmp.v17i3.5536

**Published:** 2016-05-08

**Authors:** Zhangkai J. Cheng, Regina M. Bromley, Brad Oborn, Martin Carolan, Jeremy T. Booth

**Affiliations:** ^1^ School of Physics, University of Sydney Sydney NSW Australia; ^2^ Northern Sydney Cancer Centre, Royal North Shore Hospital St Leonards NSW 2065 Australia; ^3^ Illawarra Cancer Care Centre, Wollongong Hospital Wollongong NSW 2500 Australia; ^4^ Centre for Medical Radiation Physics (CMRP), University of Wollongong Wollongong NSW 2500 Australia

**Keywords:** spine SBRT, dose calculation accuracy

## Abstract

The metallic fixations used in surgical procedures to support the spine mechanically usually consist of high‐density materials. Radiation therapy to palliate spinal cord compression can include prophylactic inclusion of potential tumor around the site of such fixation devices. Determination of the correct density and shape of the spine fixation device has a direct effect on the dose calculation of the radiation field. Even with the application of modern computed tomography (CT), under‐ or overestimation of dose, both immediately next to the device and in the surrounding tissues, can occur due to inaccuracies in the dose prediction algorithm. In this study, two commercially available dose prediction algorithms (Eclipse AAA and ACUROS), EGSnrc Monte Carlo, and GAFchromic film measurements were compared for a clinical spine SBRT case to determine their accuracy. An open six‐field plan and a clinical nine‐field IMRT plan were applied to a phantom containing a metal spine fixation device. Dose difference and gamma analysis were performed in and around the tumor region adjacent to the fixation device. Dose calculation inconsistency was observed in the open field plan. However, in the IMRT plan, the dose perturbation effect was not observed beyond 5 mm. Our results suggest that the dose effect of the metal fixation device to the spinal cord and the tumor volume is not observable, and all dose calculation algorithms evaluated can provide clinically acceptable accuracy in the case of spinal SBRT, with the tolerance of 95% for gamma criteria of 3%/3 mm.

PACS number(s): 87.53.bn, 87.53.Ly, 87.55.kd

## I. INTRODUCTION

Approximately 30% of patients with metastatic cancer will develop spinal metastases, which is a significant cause of death in patients with systemic cancer.^(1)^. Spinal cord compression is a serious complication of spinal metastasis that can produce profound disability and shortened life expectancy. Patchell et al.^(2)^ published a landmark study in 2005 which established spinal fixation followed by radiotherapy as the standard of care for spinal cord compression.

Metal implants, such as dental implants, hip prostheses, and spine fixation devices in patients, raise concerns in radiation therapy because they potentially lead to an inaccurate patient dose calculation in three ways: 1) they permit metal‐induced artifacts in the CT images; 2) they interact with the treatment beam and are poorly modeled by some dose prediction algorithms; and 3) they lead to the formation of cold and hot spots,^3^, ^4^, ^5^ which is particularly important in the image‐guided, intensity‐modulation radiation therapy (IG‐IMRT) era which combines inverse optimization with daily alignment.

Metallic spinal implants have been made of stainless steel in the past, but recently, titanium alloy has been used for spinal implants.^(6)^ While numerous studies have discussed the effects of hip prostheses and dental implants on radiation therapy, the studies discussing the effects of small‐size spinal prostheses on spinal radiation therapy are limited. Liebross et al.^(7)^ investigated the effect of spinal titanium stabilization rods on spinal cord radiation dose, and concluded that the rod can cause a 5%–10% reduction in the dose of a 6 MV beam delivered to the region directly behind the rod, but the dose perturbation was insignificant to the spinal cord lying between the rods. Pekmezci et al.^(8)^ demonstrated the dose perturbation effects of various spinal implant techniques. Meshbahi and Nejad^(9)^ performed a Monte Carlo (MC) study on the dosimetric impact of metallic spinal rods in photon beams. They showed dose reductions of approximately 6% and 11% for titanium and steel rods respectively. The study showed that the target region directly behind the rods received significantly less dose (shadow effect), while the spinal cord dose was unchanged. Son et al.^(6)^ conducted a dosimetric measurement on a pair of titanium screws in a phantom, for a set of intensity‐modulated Tomotherapy and CyberKnife plans. After comparing the measurement by an ionization chamber and Gafchromic EBT film with the result of Pinnacle Treatment Planning System (TPS) calculations, they concluded the range of errors caused by the titanium implants is beyond a clinically acceptable range. By studying the dosimetric effect of titanium implants in spinal SBRT, Wang et al.^(10)^ showed that the Pinnacle TPS with the standard CT density table can overestimate the dose by almost 6% at points when compared to the MC simulation. Li et al.^(11)^ showed that spinal internal fixation materials significantly influence dose by attenuation and backscattering, while pointing out the controversy regarding the best method to determine the correct radiation dose.

Previous studies have had limited success in quantifying the dosimetric effect of high‐Z metals on the regions that they are immediately next to, and relating this to clinical TPS algorithms. Understanding the changes in dosimetry, as well as the clinical quality of metal implants, is still in its early stages. The aim of this study is to characterize the impact of the high‐density spinal implants on the dose distribution to tissues in proximity. Treatment conditions were replicated by a specially designed phantom together with a commercially available Medtronic (Medtronic Australasia Pty. Ltd., Ryde, NSW, Australia) spine fixation device to verify dose calculations. Gafchromic film measurements were used as the gold standard to validate the dose calculated by the Eclipse (TPS) and Monte Carlo (MC) calculations in order to determine their accuracy.

## II. MATERIALS AND METHODS

### A. Spine fixation device and water phantom

**Figure 1:**
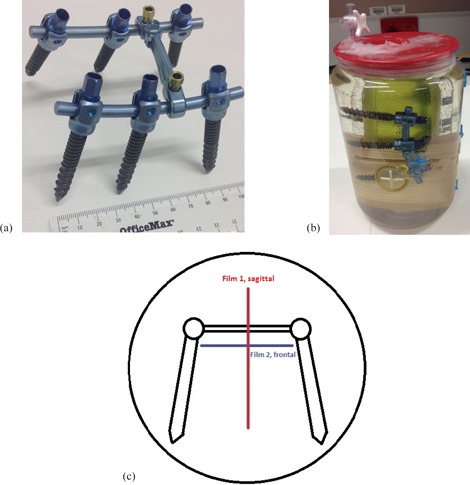
Phantom material: (a) the spine fixation device; (b) the device was submerged in water and fixed in position; (c) the overhead view of the phantom.

A spine fixation device, shown in Fig. 1(a), was utilized in this study. A sample of the device was sent to and analyzed by the University of New South Wales Analytical Centre, using the laser ablation inductively coupled plasma mass spectrometry (ICP‐MS) technique to determine the elemental composition by weight.

An in‐house water phantom was constructed to fix the device in water and allow the Gafchromic films (Ashland Inc., Covington KY) to be inserted in the sagittal and frontal orientations, as shown in Figs. 1(b) and (c). The phantom acts as a surrogate for the spinal region of a patient.

The phantom was scanned with a Philips Brilliance Big Bore CT scanner (Philips Health Care, Cleveland, OH). Images were acquired with 16‐bit depth at 50 mAs, 120 kVp, with $133\times 133\;{\text{mm}}^{2}$ FOV, 1 mm slice thickness and 1024×1024 pixels. The pixel size is hence $∼0.13\times 0.13\;{\text{mm}}^{2}$. The images were postprocessed to reduce metal artifacts using O‐MAR (Philips Health Care).

### B. Treatment plans

#### B.1 Open 6‐field plan

**Figure 2:**
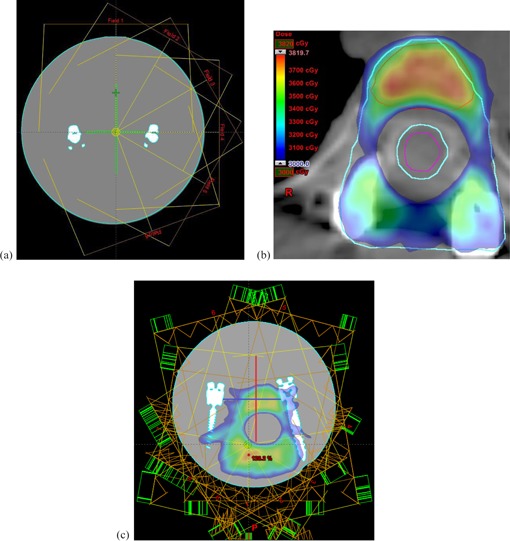
Treatment plans as viewed in Eclipse TPS: (a) six fixed beams; (b) clinical IMRT plan; (c) IMRT plan transferred to phantom. For (a) and (b), cyan contour=30 Gy PTV, red contour=35 Gy PTV, purple contour=spinal cord. For (c), red and blue lines denote the positions in which the films were fixed.

To investigate the accuracy of planning algorithms six $10\times 10\;{\text{cm}}^{2}$ static 6 MV X‐ray fields were planned and delivered, as shown in Fig. 2(a). The six square fields share a common isocenter, which was positioned on the central axis of the phantom. For each field 200 MU was delivered, and the dose was measured for both the sagittal and frontal orientation. The fields vary by the gantry angle, which are 0°, 30°, 60°, 90°, 120°, and 160°, respectively. The angles were chosen such that differing amount of the metal fixation device would shadow the beam incident on the films. Hence the variation in dose caused by the fixation device could be measured. The amounts of shadow range from 0% to ∼30% of the total area of the film, depending on the gantry angle and the film orientation.

#### B.2 IMRT plan

A clinical 6 MV IMRT plan consisting of nine fields created for a spinal metastases patient was copied and transferred onto the spine fixation device phantom, as shown in Figs. 2(b) and (c). A film was orientated in the sagittal direction to measure the dose through both the spinal cord and the tumor volume. A film was orientated in the frontal direction to measure the dose through the tumor volume. By using an actual patient plan, this experiment allows us to compare the complex 2D dose predicted by the Eclipse TPS (Varian Medical Systems, Palo Alto, CA) to the measured dose distribution using film dosimetry.

### C. AcurosXB, AAA, and Monte Carlo dose calculations

To simulate the dosimetric effects of metallic inserts, the CT scan sets were planned using Eclipse TPS (version 11.0.3). For such high‐Z materials contained in the spine fixation device even after the application of O‐MAR the metal artifacts were still present. Hence the CT images were manually contoured and Hounsfield units (HU) values were assigned to each structure (water (0), fixation device (7846), etc.). This was done by first contouring the spine fixation. The TPS contouring tools has a HU ranger function that allows the user to set an upper and lower HU limit for a contour within a given ROI. For the spine fixation, the lower HU value was set to 7846. The dimensions of the resultant contour were checked, using calipers, against physical dimension to ascertain the contour was the ground truth. The remainder of the phantom was contoured and assigned a HU value of 0 corresponding to water. The spine fixation contour was then subtracted from the larger phantom contour to create the two different HU distributions shown in Figs. 2(a) and (c), thus removing all metal artifacts. The HU value of 7846 was derived from the elemental composition determined by the University of New South Wales Analytical Centre, the HU value range for titanium metal, and the CT value obtained from CT imaging. The density information is obtained in the form of HU from CT the scan calibrated for known relative electron density $\left(\rho_{e}\right)$ materials. By calibrating a specific relationship between the CT scanner HU and $\rho_{e}$ of scanned materials, the transitional beam effective path lengths can be obtained for dosimetry purposes.^(12)^. In Eclipse, the range of HU is defined for a relative electron density (RED) range 0 to 10. In our center, the HU range is determined using the RMI electron density phantom (model 465) (Gammex RMI, Middleton, WI) set material for REDs below 1.707 corresponding to a HU of approximately 1224. To characterize the curve for REDs above 1.7, two additional metallic rods were purchased and inserted into solid water plugs to be used in the RMI phantom: a titanium core plug (CIRS 062MA‐12; CIRS, Norfolk, VA) with a RED of 4.66; and a Smootharc 3.2 mm rod (BOC, North Ryde, NSW, Australia) with a RED of 8.25 (Grade 316L‐17, with manufacturer‐stated chemical composition by weight: 65.28% Fe, 0.02% C, 0.8% Si, 0.7% Mn, 18.5% Cr, 12.0% Ni, 2.7% Mo).

Two dose calculation algorithms in Eclipse TPS were used and compared: AAA and Acuros XB (Varian Medical Systems) (both version 11.0.30). The calculation grid size was set to be 1 mm, which is the lower limit on Eclipse TPS. A clinical SBRT plan of a patient with a spine fixation device implant was transferred onto the phantom geometry. The clinical plan consists of nine IMRT 6 MV photon fields. The MU used for the patient plan was used in the phantom plan. The isocenter in the phantom plan was position such that the spinal region goes through the center of the fixation device such that the Gafchromic film would measure the dose through the spinal cord.

The CT DICOM files, RTDOSE (dose per beam and sum of prescription), RTSTRUCT file, and RTPLAN files were exported for Monte Carlo simulations with BEAMnrc/DOSXYZnrc using an in‐house system developed at the Illawarra Cancer Care Centre, Wollongong Hospital.^(13)^ The Monte Carlo dose grid size was 0.52×0.52×2 mm, which exactly matches 4 CT pixels by 1 slice. The materials simulated included air, water, and Ti. The Ti (density 4.5 g/cc) material was spatially assigned according to the contours defined in the RTSTRUCT file.

### D. Film dosimetry

Gafchromic EBT3 films were used for the measurement of 2D dose distributions. The films were scanned before and after the irradiation, and the optical density (OD) value obtained by comparing the two.

#### D.1 Calibration and irradiation

The dose calibration curves were determined by exposing small pieces of film $\left(2\times 2\;{\text{cm}}^{2}\right)$ to eight different doses ranging from 25 to 600 MUs, under a standard phantom setup of 100 SSD, 5 cm depth, $5\times 5\;{\text{cm}}^{2}$ field size, and 10 cm backscattering material. The film was placed at the central axis. The percentage depth dose (PDD) at that depth for a 6 MV beam is 85.7 with the Varian Trilogy linear accelerator. The OD to dose calibration curve was fit using a third order polynomial and was used to calculate the dose received by the actual film.

The phantom was set up according to replicate the treatment plan in Eclipse TPS described above. The films were inserted into the phantom and fixed in the sagittal and frontal orientations. The phantom was treated according to the plan.

#### D.2 Reading protocol

An Epson Perfection V700 flatbed scanner (US Epson, Long Beach, CA) was used to study the EBT3 response. The scanner had a maximum spatial resolution of 4800×9600 dpi. It employed a fluorescent light source. All films were scanned 20‐24 hrs following the irradiations. Each piece of film was scanned near the center of the scanner in the landscape orientation with the same side facing upwards. Films were scanned using EPSONSCAN software with all filters and image enhancement options turned off. A scanning resolution of 72 dpi and an imaging mode of 48‐bit RGB (16 bit per colour) were used. To minimize the warming‐up effect, at least five successive scans for warm‐up were performed at the beginning of each measurement series.^(14)^. To reduce the measurement uncertainty, each piece of film was scanned three times and each pixel value was taken from the average of three scans. The images were saved as tagged image file format (TIFF) and were then imported to ImageJ (NIH) and MATLAB R2013a (MathWorks, Natick, MA). In‐house image manipulation routines were used to extract only the green channel pixel values of the $4\times 8\;{\text{cm}}^{2}$ region of interest (ROI) of the RGB scanned image. The film data within 5 mm of each border of the films were ignored.^(15)^ To reduce inherent noise, a 2D median filter of 3×3 pixels was applied. The scanner response values were converted to OD in Microsoft Excel, in which the OD to dose calibration curve was generated. The curve was used to convert the measured planar film image to a dose image, which was then compared to the plan calculation results provided by AAA, Acuros XB, and MC simulations. The comparison of dose was performed using MATLAB and DoseLab version 6.40 (Mobius Medical Systems, Houston, TX).

### E. Gamma analysis

A comparison of the measured 2D dose distribution from the EBT3 film with dose calculated using Acuros XB, AAA, and MC was performed using a gamma analysis.^(16)^

The gamma criteria chosen in this study are 2% relative dose difference and 2 mm DTA (2%/2 mm) with a 90% tolerance, as well as 3% relative dose difference and 3 mm DTA (3%/3 mm) with a 95% tolerance. The dose is determined relative to the global maximum value within the ROI and pixels with values 20%–120% were included in the analysis. DoseLab version 6.40 was used for the analysis. The ROI was manually selected before the comparison such that the regions within 5 mm from the edges of the film were ignored.^(15)^ The shortest distance between the selected ROI and the pedicle screws is approximately 10 mm. Image alignment was performed manually in the software.

## III. RESULTS

The elemental composition of the spine fixation devices was tested using ICP‐MS technique, and the percentage by weight was determined: 90.135% Ti, 5.820% Al, 2.647% V, 0.279% Fe, 0.016% Mg, 0.016% Sn, 0.010% Br.

**Figure 3:**
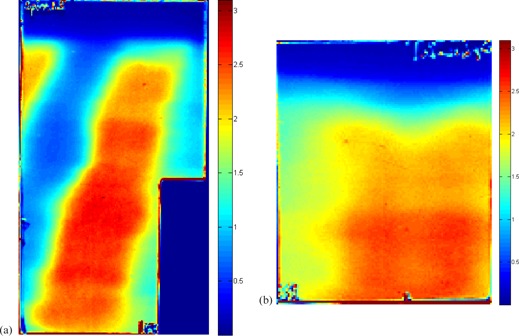
2D dose map obtained from film dosimetry: (a) Film 1, sagittal $\left(10.0\times 5.5\;{\text{cm}}^{2}\right)$; (b) Film 2, frontal $\left(5.0\times 4.0\;{\text{cm}}^{2}\right)$.

Figure 3 shows the averaged 2D film measurements for IMRT plan. Each film measurement was repeated three times, and the dose maps were aligned and averaged prior to further analysis.

### A. Gamma analysis: single fixed beams

Table 1 lists the gamma pass rate for the six single fixed beams with sagittal and frontal orientation, comparing film against AAA, Acuros XB (AXB) and MC. Only the gamma criteria of 2%/2 mm are listed for comparison. The results indicate that for fields in which a bulk part of the film is shadowed by the spine fixation device, AAA and Acuros XB display an unacceptable amount of disagreement (<90%) in dose compared to the film measurement.

**Table 1:** Gamma pass rate for IMRT plan.

	*Sagittal*		*Frontal*	
	2%/2 mm	3%/3 mm	2%/2 mm	3%/3 mm
Film vs. Acuros XB	97.9%±1.0%	100%	97.8%±1.0%	100%
Film vs. AAA	98.5%±1.0%	100%	97.8%±1.0%	100%
Film vs. MC	93.7%±2.0%	99.0%±0.5%	92.5%±3.0%	98.8%±0.5%
Acuros XB vs. AAA	99.2%±0.5%	99.8%	99.5%	99.8%

### B. Gamma analysis: IMRT plan

**Figure 4:**
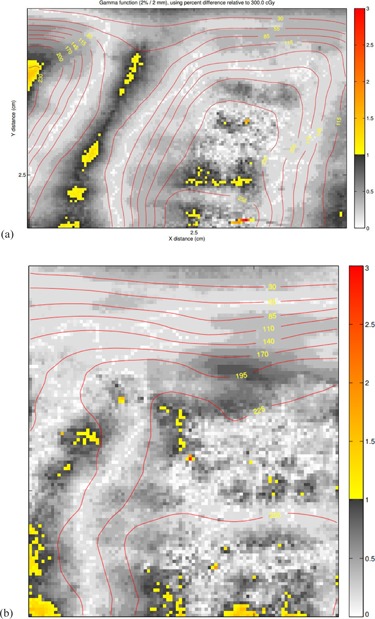
Gamma analysis result of film vs. AAA (2%/2 mm): (a) sagittal; (b) frontal.

**Figure 5:**
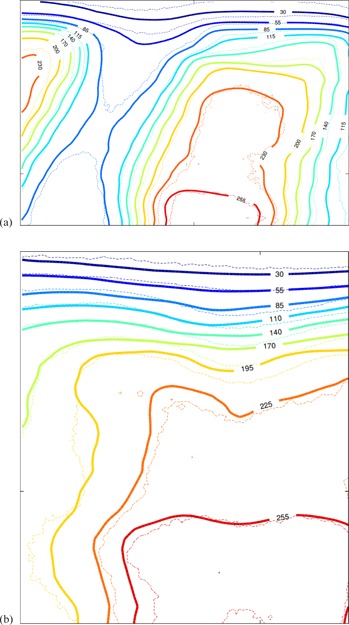
Dose contour comparison of film (thin dash) vs. AAA (thick solid): (a) sagittal. (b) frontal.

Figure 4 shows the results of gamma analysis between film dosimetry and AAA, and Fig. 5 compares the dose contours between film dosimetry and AAA. Table 2 lists the gamma pass rate of the three dose calculation algorithms against the film dosimetry, as well as comparison of Acuros XB against AAA. The uncertainty is due to the variability of the ROI selection and manual alignment. The error range was obtained upon repeating the analysis. The results show acceptable calculation accuracy for all of Acuros XB, AAA, and MC calculations (>90% with the gamma criteria of 2%/2 mm, or >90% with the gamma criteria of 3%/3 mm is deemed acceptable^(17)^). No significant difference in the dose calculation accuracy between Acuros XB and AAA was observed; however, the result of MC gives lower gamma pass rates, due to the inherent statistical noise of the dose distribution. It is expected that increasing the total number of particle histories or smoothing the dose distribution would slightly improve the gamma passing rates.

**Table 2:** Gamma pass rate for fix fields. Gamma criteria: 2%/2 mm.

*Field*		*1*	*2*	*3*	*4*	*5*	*6*
Sagittal	Film vs. AAA	98.4%	89.4%	72.1%	59.2%	79.7%	96.4%
	Film vs. AXB	98.3%	94.6%	81.7%	70.6%	95.7%	99.2%
	Film vs. MC	99.4%	98.7%	92.5%	90.3%	93.5%	92.3%
Frontal	Film vs. AAA	93.8%	89.4%	91.6%	90.9%	84.3%	96.7%
	Film vs. AXB	100%	98.8%	99.4%	97.7%	100%	98.4%
	Film vs. MC	99.2%	99.8%	100%	94.2%	94.4%	99.7%

## IV. DISCUSSION & CONCLUSION

We investigated and demonstrated quantitatively the dosimetric effect of metallic material in spine SBRT by evaluating and comparing between different dose calculation methods. This was achieved through experimental measurement of dose around the spine fixation device in multi‐beam treatment and single fixed beam treatments. The results were evaluated by gamma analysis, revealing information about the dose perturbation effect of metals and their clinical impact.

An open six‐field plan was delivered to the spine fixation device phantom, and the dose distribution was measured in two planes. The single fields that had the smallest gamma pass rate, with all three algorithms, was for field 4 in both the frontal of sagittal films. This field contained the largest ratios of overshadowing by the fixation device. From these results, AAA and Acuros XB are unable to correctly calculate the dose directly behind the spine fixation device with gamma pass rates as low as 59.2% and 70.6%, respectively. When the plan complexity and number of fields was increase for the patient case, the impact of the fixation device on the dose to the measurement planes diminishes. This is reflected in the gamma result for the IMRT patient plan where the pass rate increases significantly for both the AAA and Acuros plans. Consistency between MC and film was shown in all cases.

The gamma pass rates obtained suggests calculation inaccuracy consistent with previous findings, in which various dose calculation algorithms were shown to be unable to correctly predict the doses in close proximity to metal rods.^6^, ^9^, ^10^, ^11^ AAA failed to accurately predict the dose behind the spine hardware in four out of the six single fields. Since the ratio of spine hardware in the field was negligible for one and six, the results for the other field indicated the algorithm's inability to correctly account for spine hardware in the dose calculation. The 11% increase in gamma score for the sagittal film of field 4 would indicate the Acuros is better at predicting the dose directly behind the spine fixation device compared with AAA. Care needs to be taken with using AAA for simple beam arrangements, as the agreement with the measured dose was poor.

One possible explanation for the good agreement seen in the IMRT plan is that the spine fixation device has very little dosimetric effect in that scenario. This hypothesis was tested by overwriting the fixation device phantom with water density in Eclipse TPS, and carrying out a dose calculation with AAA (as both AAA and Acuros XB can deliver accurate dose results for a water phantom^(18)^). Gamma analysis (2%/2 mm) was used to compare the resulting 2D dose map with film dosimetry result, and pass rates of 98.3%±0.5% for sagittal and 99.5%±2.0% for frontal were obtained.

The result for the IMRT plan suggests an insignificant dose change near the spinal cord and the tumor volume when the fixation device is present, possibly due to the averaging effect of the nine‐field IMRT plan, as most of the beams do not go through the pedicle screws, as well as the diminishing dose effect with distance to the metal. By comparing dose to another water‐filled phantom, we conclude that the presence of the spine fixation device plays no significant role to the spinal cord and tumor volume dose in the IMRT.

## ACKNOWLEDGMENTS

Zhangkai Jason Cheng thanks the Medical Physics team at Northern Sydney Cancer Centre and Prof. Paul Keall for fruitful discussions around this work. The authors acknowledge the donation of the spine fixation for this study from Medtronic Australasia Pty Ltd.

## COPYRIGHT

This work is licensed under a Creative Commons Attribution 4.0 International License.

## Supporting information

Supplementary Material
