# Supplementary material for: On the accuracy of dose prediction near metal fixation devices for spine SBRT
Source: J Appl Clin Med Phys. 2016 May 8;17(3):475–85. doi: 10.1120/jacmp.v17i3.5536 (PMC5690901; doi:10.1120/jacmp.v17i3.5536)
Supplement: Supplementary file 1 — Supplementary Material [file ACM2-17-475-s001.doc]

**On the accuracy of dose prediction near metal fixation devices for spine SBRT**

**Zhangkai J Cheng^1^, Regina M Bromley^2^, Brad Oborn^3,4^, Martin Carolan^3,4^, Jeremy T Booth^2,1^**

^1^School of Physics, University of Sydney, Sydney NSW Australia

^2^Northern Sydney Cancer Centre, Royal North Shore Hospital St Leonards NSW 2065 Australia

^3^Illawarra Cancer Care Centre, Wollongong Hospital, Wollongong NSW 2500 Australia

^4^Centre for Medical Radiation Physics (CMRP), University of Wollongong, Wollongong NSW 2500 Australia
